# Yield of diagnostic tests in unexplained renal hypophosphatemia: a case series

**DOI:** 10.1186/s12882-018-1017-z

**Published:** 2018-09-04

**Authors:** A. P. Bech, E. J. Hoorn, R. Zietse, J. F. M. Wetzels, T. Nijenhuis

**Affiliations:** 1 Department of Nephrology, Radboud university medical center, Nijmegen, The Netherlands; 2 Department of Internal Medicine, Division of Nephrology and Transplantation, Erasmus medical center, Rotterdam, The Netherlands

**Keywords:** Hypophosphatemia, FGF23, Tumor-induced osteomalacia

## Abstract

**Background:**

Isolated renal hypophosphatemia may be inherited or acquired. An increasing number of patients with unexplained renal hypophosphatemia is being referred to our clinics, but the optimal diagnostic work-up is not known. Therefore, the aim of this study was to assess the diagnostic yield in these patients.

**Methods:**

We retrospectively evaluated all patients who were referred because of unexplained isolated renal hypophosphatemia to two academic tertiary referral centers in The Netherlands in the period of 2013–2017.

**Results:**

We evaluated 17 patients. In five female patients renal hypophosphatemia could be attributed to the use of oral contraceptives. The other 12 patients had a median age of 48 years (10 males). There were no other signs of tubulopathy and none of the patients used drugs known to be associated with hypophosphatemia. FGF23 levels were above normal (> 125 RU/ml) in 2/12 patients. Genetic testing, performed in all patients, did not identify a mutation in genes known to be associated with renal phosphate wasting. A scan with a radiolabeled somatostatin analogue was performed in 8 patients. In one patient, with an FGF23 level of 110 RU/ml, an increased uptake of the somatostatin analog was observed due to tumor induced osteomalacia (TIO).

**Conclusions:**

Oral contraceptive use is an important but under-recognized cause of renal hypophosphatemia. The cause of isolated renal hypophosphatemia remained unexplained in the majority of other patients despite extensive and expensive additional investigations. The pre-test probability for tumor-induced osteomalacia or inherited renal hypophosphatemia in a patient with aspecific complaints and a normal FGF23 level is low. Further research is needed to investigate which patients should be screened for TIO. At present we suggest to perform somatostatin scans only in patients with severe complaints, elevated FGF23 levels, or progressive disease.

## Background

Phosphate is freely filtered by the glomerulus and 80–90% is reabsorbed at the brush border membrane of the proximal tubule, through apical sodium-dependent phosphate transporters (primarily NaPi2a and NaPi2c). The principal regulators of this transcellular phosphate reabsorption are dietary phosphate, parathyroid hormone (PTH), 1,25 dihydroxy vitamin D_3_ (1,25OHD_3_) and fibroblast growth factor 23 (FGF23). A defect in one of these transporters or perturbation in one of the regulating factors can result in increased renal phosphate loss. Various inherited phosphate wasting disorders have been identified, including X-linked autosomal hypophosphatemia (XLH; due to a mutation in PHEX), autosomal dominant hypophosphatemic rickets (ADHR; due to impaired cleavage of FGF23 caused by a mutation in FGF23), autosomal recessive hypophosphatemic rickets (ARHR; due to a mutation in DMP1) and Fanconi renotubular syndrome-2 (dysfunction of NaPi2a due to homozygous mutations in *SLC34A1*) [1]. Some of these inherited disorders, such as ADHR, can become apparent after childhood, which can make the differentiation with acquired disorders difficult. The most common causes of acquired isolated renal hypophosphatemia are drugs (for example acetazolamide, bisphosphonates, diuretics, glucocorticosteroids, imatinib, acyclovir, aminoglycosides, tenofovir and valproic acid), hyperparathyroidism and tumor induced osteomalacia (TIO) [2]. TIO is a rare disorder in which there is renal phosphate wasting due to tumors that secrete phosphaturic factors (most commonly FGF23) [3–5].

The cause of hypophosphatemia is often identified during the initial diagnostic evaluation of patients presenting with (renal) hypophosphatemia. Yet, an increasing number of patients is being referred to our clinics because of unexplained chronic renal hypophosphatemia. The optimal diagnostic work-up of these patients is not known. We report the diagnostic yield of extensive evaluation in these patients.

## Methods

We retrospectively evaluated all patients who were referred because of unexplained chronic renal hypophosphatemia,which was confirmed on several occasions, to two university hospitals in The Netherlands in the period of 2013–2017. In these patients, well-known causes of acquired renal hypophosphatemia had been previously excluded. Renal hypophosphatemia was defined as a serum phosphate below 0.70 mmol/l and an inappropriately high renal phosphate excretion. Fractional excretion of phosphate was calculated as (urine phosphate * serum creatinine)/(serum phosphate * urine creatinine). TmP/GFR was calculated by the method of Bijvoet [6]. All measurements were performed during low serum phosphate levels. The reference value for serum phosphate in our centers is 0.80–1.40 mmol/l (variation coefficient of 3.7%). In most patients a fasting serum phosphate was measured at 8.00 am because it is known that serum phosphate shows a circadian rhythm with the lowest value between 8.00–11.00 am [7, 8]. To exclude that the hypohosphatemia was due to this circadian rhythm, we determined serum phosphate in 100 fasting anonymous samples of patients visiting the outpatient clinic with an eGFR > 60 ml/min/1.73 m^2^, withdrawn between 8.00–10.00 am. The mean serum phosphate level was 1.06 mmol/l (SD 0.17, range 0.67–1.42 mmol/l), indicating that the hypophosphatemia in our patients was not the result of the early morning blood sampling.

General (proximal) tubular dysfunction was assessed by measuring glucose, pH, urate and α1-microgobulin in urine. Glucose, calcium, potassium, creatinine (enzymatic method) and phosphate were measured on a Cobas c6000 (Roche, Switzerland) or c18000 (Abbott, USA) analyzer.

The α1-microgobulin concentration in urine was measured on a BNII nephelometer (Siemens, Germany) and urinary pH was measured with a PHM220 potentiometer (Hach Lange Nederland, the Netherlands). Genetic testing for mutations in genes that are known to be associated with renal phosphate wasting (*DMP1, FGF23, FGFR1, GALNT3, PHEX, SLC34A1, SLC34A3, SLC9A3R1*) was done by Sanger sequencing and MPLA on genomic DNA derived from peripheral blood cells. Vitamin D was measured by LC-MSMS. Intact PTH (second generation) was measured by an ECLIA on a modular E170 analyzer (Roche, Switzerland). FGF23 was measured by an ELISA of Immutopics measuring both intact FGF23 and C-terminal fragments, San Clemente, CA, USA. Somatostatin scans were performed per protocol in all patients with an elevated FGF23 level. In addition, somatostatin scans were performed in some patients with a normal FGF23 level at the discretion of the treating physician. These scans use somatostatin analogs (pentetreotide, TOC, TATE or NOC), which are labelled with tracer molecules (^111^Indium, ^68^Gallium or ^99^ Technetium) and sometimes a chelator molecule (DOTA). The somatostatin scans that were used in our clinics are the ^68^Ga-DOTA-TOC PET/CT scan and the ^111^Indium-pentetreotide SPECT/CT scan.

## Results

Seventeen patients with unexplained chronic renal hypophosphatemia were evaluated. The median age was 42 years old, 10 patients were male and there were no other signs of tubulopathy in any of these patients.

**Fig. 1:**
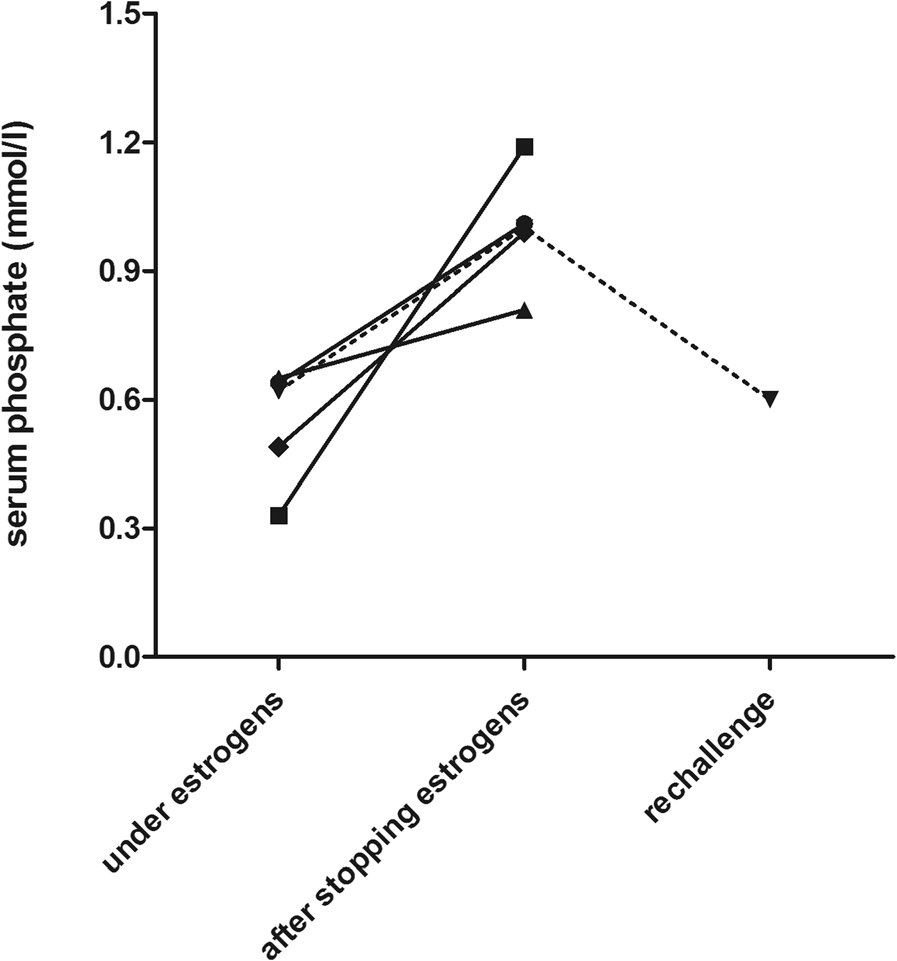
Course of serum phosphate levels before and after stopping oral contraceptives in 5 women

Five female patients used oral contraceptives, containing estrogens, at the time of referral. Their demographical and clinical characteristics are shown in Table 1. After discontinuing the oral contraceptives, renal hypophosphatemia resolved in all patients with normalisation of serum phosphate levels (Fig. 1). TmP/GFR was measured in three patients before and after stopping the oral contraceptives and increased from 0.58 mmol/l (range 0.30–0.62) to 0.92 mmol/l (range 0.78–0.95). A re-challenge with the oral contraceptive was performed in one patient after which renal hypophosphatemia re-emerged. Characteristics of the other 12 patients are shown in Table 1. PTH was above normal (8.7 pmol/l) in only one patient. This patient did not have vitamin D deficiency and calcium excretion was normal (5 mmol/day). One patient had a 25-hydroxyvitamin D (25OHD) level below 50 nmol/l together with a slightly elevated 1,25OHD_3_ level of 206 pmol/l. 1,25OHD_3_ was above normal in another four patients and FGF23 was above 125 RU/ml in two patients.

**Table 1:** Patient characteristics

	Oral contraceptive induced hypophosphataemia (*N* = 5)	Unexplained renal hypophosphataemia (*N* = 12)	Reference value
Age (years)	34 (28–53)	48 (24–64	
Gender (M/F)	0/5	10/2	
Serum phosphate (mmol/l)	0.62 (0.33–0.65)	0.59 (0.36–0.77)	0.80–1.40
TmP/GFR (mmol/l)	0.58 (0.30–0.68)	0.50 (0.38–0.55)	0.80–1.40
FePi (%)	11 (4–13)	18 (5–35)	< 5
Serum creatinine (μmol/l)	74 (62–77)	87 (53–142)	60–110
eGFR (ml/min/1.73m^2^)^a^	95 (80–118)	95 (45–122)	> 90
Serum calcium (mmol/l)	2.27 (2.20–2.33)	2.39 (2.27–2.59)	2.20–2.65
serum potassium (mmol/l)	3.9 (3.7–4.4)	3.9 (3.5–4.5)	3.5–4.7
Serum magnesium (mmol/l)	0.80 (0.79–0.91)	0.82 (0.78–0.95)	0.70–1.10
PTH (pmol/l)	5.3 (4.7–6.6)	5.2 (3.2–8.7)	1.0–6.5
25OHD(nmol/l)	106 (57–148)	72 (40–135)	> 50
1,25OHD_3_ (pmol/l)	169 (129–246)	140 (78–322)	50–150
cFGF23 (RU/ml)	66 (48–124)	89 (53–202)	< 125
24 h urinary calcium excretion (mmol)	3 (2.2–4.8)	5.5 (0.6–11.0)	< 5.0

Values are given as median (range)

FePi = fractional excretion of phosphate

^a^eGFR was calculated by CKD-EPI formula

Genetic testing, which was performed in all remaining patients, did not show any mutation in genes known to be associated with renal phosphate wasting.

The two patients with an FGF23 level above 125 RU/ml both underwent an ^111^Indium-pentetreotide SPECT/CT. These scans did not show increased somatostatin uptake that would have been suggestive of TIO. Three additional patients, with normal FGF23 levels, underwent an ^111^Indium-pentetreotide SPECT/CT and three additional patients a ^68^Ga-DOTA-TOC PET/CT scan, at the discretion of the treating physician. One of these six scans, a ^68^Ga-DOTA-TOC PET/CT scan, showed increased uptake in the right lateral femur suggestive of TIO. This patient had a normal serum FGF23 level of 110 RU/ml but progressive hypophosphatemia which required increasing doses of phosphate replacement. In this patient the tumor in the femur was resected (histology showed an enchondroma) after which renal hypophosphatemia resolved.

## Discussion

Our study shows that in female patients presenting with previously unexplained renal hypophosphatemia, estrogen-induced hypophosphatemia due to oral contraceptive use appears to be frequent. In 5 out of 7 female patients the hypophosphatemia could be attributed to the use of oral contraceptives. This is a relatively unknown and often overlooked cause of renal phosphate loss. No previous study reported hypophosphatemia as the result of oral contraceptive use, but a decrease in serum phosphate has been described in response to estrogen replacement therapy in postmenopausal women [9–12]. This decrease in serum phosphate was accompanied by a decrease in TmP/GFR, confirming renal phosphate wasting [11, 12]. The estrogen content of the replacement therapy that was used in these postmenopausal women was higher than the estrogen content in oral contraceptives in our patients (50 μg to 62.5 μg compared to 30 μg). Treatment with even higher doses of diethylstilbestrol diphoshate in male patients with metastatic prostate cancer also resulted in lowering of serum phosphate in 16 out of 18 patients [13]. Urinary parameters were measured in three of these patients, and were compatible with increased renal phosphate loss. Thus, the pathophysiological mechanism of the renal phosphate loss with oral contraceptive use is most likely related to the estrogen content. Indeed, estrogen treatment in ovariectomized rats and mice results in renal hypophosphatemia. In these animals both NaPi2a en NaPi2c in the proximal tubule were shown to be down regulated [14–16].

In 11/12 of the remaining patients the underlying cause of renal hypophosphatemia remained unknown, despite extensive diagnostic evaluation. Although genetic testing did not reveal a mutation, this does not rule out genetic causes as we might still have missed mutations of which the significance is not yet understood.

One patient was diagnosed with TIO. This diagnosis was made after this patient underwent a somatostatin scan, despite a normal serum level of FGF23, because he suffered from progressive hypophosphatemia.

Although most reported patients with TIO have clearly elevated serum FGF23 levels, fourteen TIO patients with normal FGF23 levels have been described in literature previously [17–25]. In these patients another phosphaturic hormone could be involved or FGF23 levels could have been misinterpreted due to the use of incorrect reference values and/or FGF23 assays.

Intact FGF23 (iFGF23) is degraded by proteolytic cleavage into inactive N- and C-terminal fragments [26, 25]. Since iFGF23 is rapidly degraded ex-vivo, special sampling procedures are required with the use of protease inhibitors [27]. In routine clinical practice in our country, a commercially available assay is used that measures both intact FGF23 and the C-terminal fragments. Therefore, we cannot exclude that some patients might have had increased iFGF23 values in the presence of normal cFGF23 values. Furthermore, reference values are based on studies in healthy volunteers. Ideally, FGF23 values in our patients with hypophosphatemia should be compared to FGF23 values in persons with FGF23-independent hypophosphatemia. Although iFGF23 is decreased in such patients [17, 28, 29], validated reference values of FGF23 adjusted for severe phosphate deficit do not exist.

We could have missed TIO’s because a somatostatin scan was not performed in all patients and because of low sensitivity of the scanning technique. Scans with radiolabeled somatostatin analogs are being used for the diagnosis of TIO as these tumors are of mesenchymal origin and frequently express somatostatin receptors (mostly SSTR 2 and 5) [22, 30–32]. The scans differ in affinity for SSTR’s and spatial resolution. PET-based imaging has better spatial resolution and signal-to-noise ratios as compared to SPECT-based imaging. The somatostatin analogues also differ in their affinity for various somatostatin receptor subtypes. DOTA-conjugated peptides have 4–8 higher affinity to SSTR2 than ^111^Indium-pentetreotide [22, 33, 31]. DOTANOC has affinity for a wide range of receptors like SSTR1, 2, 3 and 5, while DOTATATE and DOTATOC bind more avidly to SSTR2 [34, 35]. The exact percentage of SSTR positive tumours in TIO is not known, neither is the exact sensitivity of the different scanning techniques for diagnosing TIO.

Since tumors causing TIO typically are slow growing neoplasms that are benign in behaviour, a defensive diagnostic approach seems justified. We therefore do not advocate to immediately perform a somatostatin scan in all patients with unexplained chronic renal hypophosphatemia. We suggest to perform somatostatin scans only in patients with severe complaints including bone pain and fractures, elevated FGF23 levels, progressive disease and/or increasing FGF23 levels.

Another striking finding is the male predominance of the group of patients with an unexplained renal hypophosphatemia. This raises the question whether endogenous testosterone, in analogy to exogenous estrogen, plays a role in the phosphate loss. Supporting this theory is the negative correlation between serum testosterone and serum phosphate in men in the general population [36]. Data about the effect of testosterone therapy on serum phosphate is conflicting [37, 38] but testosterone depletion (states) seems to induce an increase in serum phosphate due to increased tubular phosphate re-absorption [39–41]. We did not test whether serum testosterone levels were elevated in our patients.

A main limitation of our paper is the retrospective study design, in which the analyses were performed to the discretion of the treating physician. Another limitation is the lack of other proximal tubular function markers such as aminoaciduria, which could be helpful to differentiate isolated renal hypophosphatemia from a more general proximal tubular defect as seen in Fanconi syndrome.

## Conclusions

In conclusion, oral contraceptive use is an important but under-recognized cause of isolated chronic renal hypophosphatemia. The cause of chronic renal hypophosphatemia remained unexplained in the majority of other patients despite extensive and expensive additional investigations. The pre-test probability for TIO or inherited renal hypophosphatemia in a patient with aspecific complaints and a normal FGF23 level is low. Further research is needed to investigate which patients should be screened for TIO with measurements of both cFGF23 and iFGF23 and DOTA-conjugated SPECT scans. At present we suggest to perform somatostatin scans only in patients with severe complaints including bone pain and fractures, elevated FGF23 levels, progressive disease and/or increasing FGF23 levels. This strategy seems justified in view of the benign course of TIO.

## Abbreviations

ADHR: Autosomal dominant hypophosphatemic rickets
ARHR: Autosomal recessive hypophosphatemic rickets
FGF23: Fibroblast growth factor 23
MPLA: Multiplex ligation-dependent probe amplificiation LC-MSMS Liquid chromatography mass spectrometry
PTH: Parathyroid hormone
SSTR: Somatostatin receptors
TIO: Tumor induced osteomalacia
TMP/GFR: Ratio of renal maximum tubular reabsorption of phosphate to GFR
XLH: X-linked autosomal hypophosphatemia
